# Effect of intravenously administered norepinephrine on early postoperative tissue perfusion of radial free forearm flaps in the head and neck region – retrospective analysis in the context of continuous flap monitoring

**DOI:** 10.1186/s12871-026-04169-0

**Published:** 2026-08-11

**Authors:** Mark Ooms, Philipp Winnand, Marius Heitzer, Nils Vohl, Marie Sophie Katz, Johannes Bickenbach, Frank Hölzle, Ali Modabber

**Affiliations:** 1 Department of Oral and Maxillofacial Surgery, University Hospital RWTH Aachen, Pauwelsstraße 30, Aachen, 52074 Germany; 2 Department of Intensive Care Medicine, University Hospital RWTH Aachen, Pauwelsstraße 30, Aachen, 52074 Germany

**Keywords:** Free flap, Flap perfusion monitoring, Norepinephrine, Blood flow, Oxygen saturation, Flow resistance

## Abstract

**Background:**

Postoperative flap monitoring following microvascular head and neck reconstruction can be conducted by continuous flap tissue perfusion measurement with an attached surface probe using the Oxygen-to-see (O2C) analysis system. However, intravenously administered norepinephrine may influence flap tissue perfusion and affect the reliability of predefined absolute thresholds used for the detection of vascular flap compromise. This study aimed to evaluate the association between early postoperative flap tissue perfusion and norepinephrine in radial free forearm flaps (RFFF).

**Methods:**

Data of patients undergoing head and neck reconstruction with an RFFF from 2020 until 2022 were retrospectively analyzed. Flap tissue perfusion was measured continuously for 8 h postoperatively with an attached surface probe using the O2C analysis system. Intravenously administered norepinephrine dose and blood flow and hemoglobin oxygen saturation as well as flow resistance (ratio of mean arterial blood pressure and flap blood flow) were analyzed for associations.

**Results:**

The study included 12 patients (5 men and 7 women). Blood flow and hemoglobin oxygen saturation were not associated with norepinephrine (B=-110.509, *p* = 0.583 and B=-12.500, *p* = 0.912). In addition, flow resistance was not associated with norepinephrine (B = 0.292, *p* = 0.825).

**Conclusions:**

Early postoperative tissue perfusion of RFFFs, measured during continuous flap monitoring with attached surface probes for the O2C analysis system, showed no association with norepinephrine with regard for blood flow and hemoglobin oxygen saturation. This supports the reliability of absolute thresholds used to detect vascular flap compromise during continuous postoperative monitoring of RFFFs with the O2C analysis system, albeit additional studies with substantial sample sizes and longer monitoring intervals are required to validate these findings.

## Background

Radial free forearm flaps (RFFF) are commonly used for reconstruction of the head and neck region offering high success rates [1, 2]. However, as RFFFs initially depend on patent microvascular anastomosis, postoperative flap monitoring is needed to early detect and correct vascular flap compromise and prevent irreversible flap failure [3–5].

In this context, technical methods are often used for postoperative flap monitoring to overcome limitations of standard clinical flap monitoring, such as the need for clinical experience and the time delay between the onset and clinical signs of vascular flap compromise; the latter being particularly detrimental as an extended time span between the occurrence and intervention for vascular flap compromise negatively affects flap salvage [4, 6, 7]. Among these technical methods is the Oxygen-2-See (O2C) analysis system, which can be used with attached surface probes for continuous flap monitoring and is based on the measurement of flap tissue perfusion compared to predefined thresholds indicating vascular flap compromise, e.g., absolute thresholds for blood flow and hemoglobin oxygen saturation [3, 8–10]. The measurement of flap tissue perfusion relies on laser Doppler spectroscopy (830 nm) to determine flap blood flow (AU [arbitrary units]) and white light spectroscopy (500–800 nm) to determine hemoglobin oxygen saturation (%), with the measured values compared to absolute cut-off values (blood flow < 60 AU and hemoglobin oxygen saturation < 40% for RFFFs) to detect vascular flap compromise with high accuracy (> 97%) [8–10].

However, the postoperative hemodynamic management of patients undergoing microvascular reconstruction of the head and neck region often involves the intravenous administration of vasopressors, e.g., norepinephrine, potentially affecting flap tissue perfusion, e.g., by altering blood pressure or vessel diameters as major determinants of tissue perfusion [11–14]. One study found no effect of norepinephrine on early postoperative tissue perfusion of microvascular free flaps based on continuous measurement with the O2C analysis system; however, that study included patients with flaps located also outside the head and neck region and focused on flap types other than RFFFs [15]. Thus, drawing conclusions for RFFFs in the head and neck region regarding the exclusion of an influence of intravenously administered norepinephrine on early postoperative flap tissue perfusion and consequently a confounding effect of intravenously administered norepinephrine during continuous early postoperative flap monitoring, based on absolute thresholds used for the detection of vascular flap compromise, appear to be limited.

This study was conducted to examine the effect of intravenously administered norepinephrine on early RFFF tissue perfusion in the head and neck region in the context of continuous postoperative flap monitoring.

## Methods

### Study population

This retrospective pilot study belongs to a project investigating the perioperative impact of blood pressure and intravenously administered medication on tissue perfusion in free flaps in the head and neck region and was approved by the local ethics committee of the Medical Faculty at RWTH Aachen University (EK 22–358; 2022-10-25) [16, 17]. All procedures complied with the relevant regulations and guidelines, including the Declaration of Helsinki.

The study included patients who underwent microvascular reconstruction of the head and neck region with an RFFF in our Department of Oral and Maxillofacial Surgery from 2020 until 2022 after resection of squamous cell carcinoma, sarcoma, adenocarcinoma, or Merkel cell carcinoma. Inclusion criteria were reconstruction with a RFFF, continuously conducted postoperative flap monitoring and intravenous administered norepinephrine, comprehensive data sets, and an age above 18 years. Exclusion criteria were reconstruction with a flap other than an RFFF, discontinuously conducted postoperative flap monitoring and intravenous administered norepinephrine, incomplete data sets, and an age equal to or below 18 years.

Surgical interventions were done under general anesthesia. The flaps were harvested without inclusion of the cephalic vein. The flap locations were intraoral the tongue, the floor of the mouth, or the mandible after bone resection and extraoral the buccal, infraorbital, or orbital region. The anastomoses were performed with the facial, lingual, or superior thyroid artery in an end-to-end configuration and with the internal jugular vein, a combination of the internal jugular vein and anterior jugular vein or vena comitans of the hypoglossal nerve, or the facial vein or retromandibular vein in an end-to-end or end-to-side configuration. Postoperatively, all patients were monitored in the intensive care unit under mechanical ventilation and analgosedation.

The clinical data were taken from the medical records. Surgery duration and flap ischemia duration were defined as described before [16]. The systemic blood pressure data spanning the first eight postoperative hours were taken from the IntelliSpace Critical Care and Anesthesia ICCA data management system (Philips Medizin Systeme Boeblingen GmbH, Boeblingen, Germany) and had been measured invasively with an arterial catheter at 5-minute intervals using the IntelliVue X2 M3002A device (Philips Medizin Systeme Boeblingen GmbH, Boeblingen, Germany) as described before [16, 17]. Mean arterial blood pressure values were calculated as follows: mean arterial blood pressure = diastolic blood pressure + 1/3 x (systolic blood pressure – diastolic blood pressure) [18].

### Data for administered norepinephrine dose

The intravenously administered norepinephrine doses during the first 8 h postoperatively (to provide hemodynamic stability) were extracted from the IntelliSpace Critical Care and Anesthesia ICCA data management system (Philips Medizin Systeme Boeblingen GmbH, Boeblingen, Germany). The doses were calculated as µg/min per kg body weight.

### Data for flap tissue perfusion

**Fig. 1:**
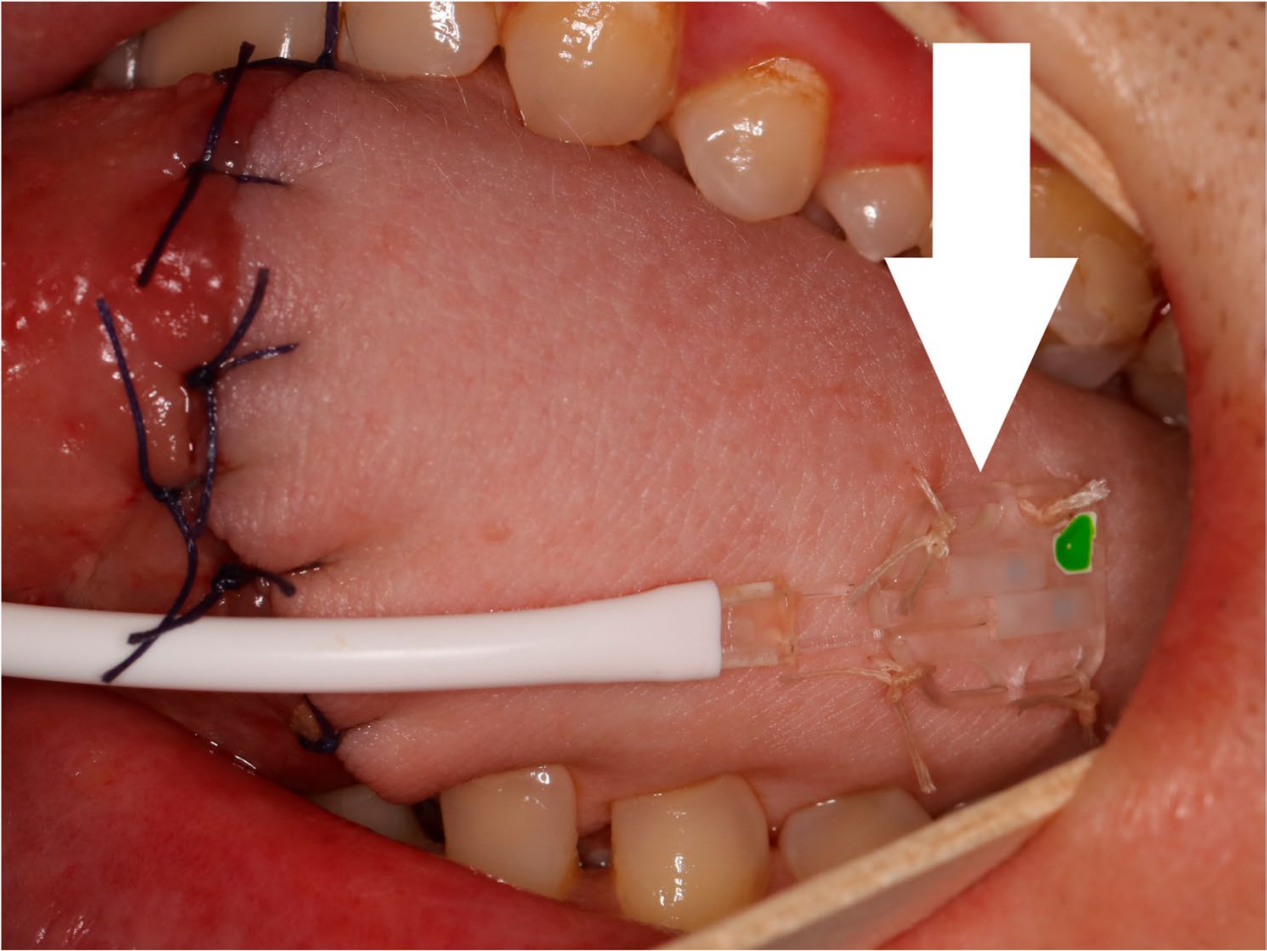
Measurement of flap tissue perfusion. Measurement of tissue perfusion of a radial free forearm flap with an attached surface probe (white arrow) in a patient reconstructed in the left tongue region

The flap tissue perfusion values spanning the first 8 h postoperatively were taken from the O2C analysis system (O2C Oxygen-to-see type, LEA Medizintechnik, Giesen, Germany) and had been measured based on Doppler and white light spectroscopy at a 3 mm tissue depth using an attached surface probe sutured in the center of the flap (Fig. 1) as described before [8, 16, 17, 19]. Flap flow resistance values were calculated as the ratio of mean arterial blood pressure and flap blood flow [20]. 

### Statistical analysis

Data were reported as numbers (with percentage) for categorical data or medians (with interquartile range) for metric data. The administered norepinephrine dose and flap tissue perfusion values were evaluated as mean values over 15 min intervals (32 intervals per patient during a 8 h measurement period). Associations between measured blood flow, hemoglobin oxygen saturation, and calculated flap flow resistance values and administered norepinephrine doses were analyzed using linear mixed models for the entire monitoring period (*n* = 384; 12 patients with each 32 intervals) and for an early and late monitoring period separately (each *n* = 192; 12 patients with each 16 intervals). Values of p were two-tailed and *p* < 0.05 was used to determine statistical significance. SPSS version 28 (SPSS, IBM, New York, NY, USA) was used for the statistical analysis.

## Results

### Clinical characteristics of the study population

The study population consisted of 12 patients, 5 (41.7%) men and 7 (58.3%) women (Table 1). The diagnoses were squamous cell carcinoma (*n* = 9), sarcoma (*n* = 1), adenocarcinoma (*n* = 1), and Merkel cell carcinoma (*n* = 1). The flap location was intraoral in 8 (66.7%) patients (tongue *n* = 3 [25.0%], floor of mouth *n* = 2 [16.7%], or mandible *n* = 3 [25.0%]) and extraoral in 4 (33.3%) patients (buccal region *n* = 2 [16.7%], infraorbital region *n* = 1 [8.3%], or orbital region *n* = 1 [8.3%]). The median RFFF size was 37 cm² (minimum 15 cm²; maximum 56 cm²; interquartile range 19 cm²). The median norepinephrine dose was 0.073 µg/min per kg body weight (minimum 0.002 µg/min per kg; maximum 0.214 µg/min per kg; interquartile range 0.064 µg/min per kg).

**Table 1:** Clinical characteristics of the study population

Number	12
*Sex* *(n)*	
male	5 (41.7%)
female	7 (58.3%)
*Age* *(years)*	72.5 (26.0)
*BMI* *(kg/m²)*	25.8 (9.0)
*ASA* *(n)*	
1 + 2	4 (33.3%)
3 + 4	8 (66.7%)
*Flap location* *(n)*	
intraoral	
tongue	3 (25.0%)
mouth floor	2 (16.7%)
mandibula	3 (25.0%)
extraoral	
buccal	2 (16.7%)
infraorbital	1 (8.3%)
orbital	1 (8.3%)
*Arterial anastomosis recipient vessel* *(n)*	
facial artery	7 (58.3%)
lingual artery	1 (8.3%)
superior thyroid artery	4 (33.3%)
*Venous anastomosis recipient vessel* *(n)*	
internal jugular vein	8 (66.7%)
internal jugular vein + other vein	2 (16.7%)
other vein	2 (16.7%)
*Surgery duration* (min)	508.5 (143.0)
*Flap ischemia duration* (min)	81.5 (22.0)

Parameters are indicated as numbers (with percentage) for categorical data (sex, ASA, flap location, arterial anastomosis recipient vessels, venous anastomosis recipient vessel) or median (with interquartile range) for metric data (age, BMI, surgery duration, flap ischemia duration); internal jugular vein + other = internal jugular vein and anterior jugular vein or internal jugular vein and vena comitans of hypoglossal nerve; other vein = facial vein or retromandibular vein; abbreviations: BMI=body mass index, ASA=American Society of Anesthesiologists score

### Association between blood flow and norepinephrine dose

Blood flow was not associated with the norepinephrine dose during the entire monitoring period (B=-110.509, *p* = 0.583) (Table 2). In addition, blood flow was also not associated with the norepinephrine dose during the early monitoring period and the late monitoring period (B=-229.917, *p* = 0.059 and B=-66.459, *p* = 1.000) (Table 2).

**Table 2:** Association between flap tissue perfusion parameters and norepinephrine

Time interval	Blood flow		Hemoglobin oxygen saturation		Flow resistance	
	B	*p*-value	B	*p*-value	B	*p*-value
*1–8*	-110.509	0.583	-12.500	0.912	0.292	0.825
*1–4*	-229.917	0.059	-52.490	0.792	1.072	0.914
*5–8*	-66.459	1.000	6.268	0.980	-0.321	0.842

Data presented as B coefficient (with p-value) corresponding to linear mixed models separately calculated for testing the association between norepinephrine and blood flow, hemoglobin oxygen saturation or flow resistance (calculated as ratio of mean arterial blood pressure and blood flow) for the entire monitoring period (1–8 h postoperatively) (*n* = 384), for the early monitoring period (1–4 h postoperatively) (*n* = 192), and for the late monitoring period (5–8 h postoperatively) (*n* = 192)

### Association between hemoglobin oxygen saturation and norepinephrine dose

Hemoglobin oxygen saturation was not associated with the norepinephrine dose during the entire monitoring period (B=-12.500, *p* = 0.912) (Table 2). In addition, hemoglobin oxygen saturation was also not associated with the norepinephrine dose during the early monitoring period and the late monitoring period (B=-52.490, *p* = 0.792 and B = 6.268, *p* = 0.980) (Table 2).

### Association between flap flow resistance and norepinephrine dose

Flap flow resistance was not associated with the norepinephrine dose during the entire monitoring period (B = 0.292, *p* = 0.825) (Table 2). In addition, flap flow resistance was also not associated with the norepinephrine dose during the early monitoring period and the late monitoring period (B = 1.072, *p* = 0.914 and B=-0.321, *p* = 0.842) (Table 2).

## Discussion

In this study, the relationship between early postoperative flap tissue perfusion of RFFFs and intravenously administered norepinephrine was investigated in the context of continuous flap monitoring with the O2C analysis system after microvascular reconstruction in the head and neck region, as an influence of intravenously administered norepinephrine on flap tissue perfusion may confound flap monitoring with regard to predefined absolute thresholds used to detect vascular flap compromise [3, 8–10, 12, 13].

The O2C analysis system enables postoperative monitoring of RFFFs by measuring flap tissue perfusion compared to predefined absolute thresholds indicative for vascular flap compromise, i.e., blood flow < 60 AU and hemoglobin oxygen saturation < 40% when used with attached surface probes measuring at 3 mm tissue depth [3, 8–10]. Concomitantly, the postoperative hemodynamic management of patients after microvascular head and neck reconstruction often involves the intravenous administration of norepinephrine, which might influence flap tissue perfusion and affect the validity of these thresholds [10–14]. However, an influence of intravenously administered norepinephrine on early postoperative tissue perfusion of RFFFs in the head and neck region remains unknown, as one previous study addressing the topic included both patients with flaps other than RFFFs and patients with flaps also located outside the head and neck region [15]. In addition, the measurement depth of the surface probe used in that study was different from the measurement depth of the surface probe used in this study [15]. Therefore, this study aimed to investigate the influence of intravenously administered norepinephrine on tissue perfusion of RFFFs located in the head and neck region during continuous early postoperative flap monitoring using an attached surface probe for the O2C analysis system with a measurement depth of 3 mm both to evaluate a potential confounding effect of intravenously administered norepinephrine and to gain further insights into the still incompletely understood physiology of microvascular free flaps regarding the influence of vasopressors on tissue perfusion [2, 10, 21].

This study did not examine a confounding effect of intravenously administered norepinephrine on continuous postoperative flap monitoring with the O2C analysis system in terms of decision making to revise a flap directly, as flap revision rates and flap failure rates in microvascular head and neck reconstruction are generally low [2].

This study revealed that flap tissue perfusion of RFFFs was not associated with intravenously administered norepinephrine in terms of blood flow, hemoglobin oxygen saturation, and flap flow resistance.

These findings are consistent with the results of an earlier study that retrospectively examined the relationship between the flap tissue perfusion of RFFFs (blood flow and hemoglobin oxygen saturation measured with the O2C analysis system), and the flap flow conductance of RFFFs (calculated as the ratio of flap blood flow to mean arterial pressure and thus as the inverse of flow resistance) with the vasopressor dose (intravenously administered norepinephrine), and found no influence of norepinephrine on flap tissue perfusion and flap flow conductance of RFFFs [22]. However, both studies are only comparable to a limited extent, as the earlier study differed from the present study in terms of the depth of flap tissue perfusion measurement (2 and 8 mm tissue depth compared to 3 mm tissue depth), with the measurement depth influencing flap tissue perfusion parameters, as well as the timing of flap tissue perfusion measurement (once intraoperatively and once postoperatively the next morning after surgery compared to continuous postoperative measurement after surgery), with the timing of flap tissue perfusion measurement also influencing flap tissue perfusion parameters [10, 16, 23]. Further, the findings of the present study are consistent with another earlier study, that determined tissue perfusion of microvascular free flaps also using the O2C analysis system and found that blood flow and hemoglobin oxygen saturation were not affected by intravenously administered norepinephrine [15]. However, that earlier study included patients with flaps that were also located outside the head and neck region, with different body regions potentially influencing flap tissue perfusion, did not focus an RFFFs, with different flap types showing different flap tissue perfusion values, and used a surface probe for the O2C analysis system that measured flap tissue perfusion in a different depth than in the present study [15, 24].

Given the generally assumed higher sensitivity of microvascular free flaps to vasopressors due to denervation after flap pedicle dissection with the pronounced vasoconstrictive effect leading theoretically to lower flap tissue perfusion, the lack of an association between tissue perfusion and intravenously administered norepinephrine in RFFFs could hypothetically be due to a lower sensitivity of RFFFs to norepinephrine, probably linked to their vascular anatomy with multiple perforator vessels that act as parallel flow resistances and increase vessel diameter from the pedicle to the microcirculation, and an outweighing of the vasoconstrictive effect (theoretically decreasing tissue perfusion) by the blood pressure raising effect (theoretically increasing tissue perfusion) of norepinephrine [8, 12, 13, 20, 25–28]. However, the present study design precludes drawing conclusions about the underlying mechanisms.

Despite the strengths of the study, with its focus on RFFFs in the head and neck region, which improves comparability between patients, there remain limitations in several aspects. Besides the small number of patients and the short time interval of flap tissue perfusion measurement analyzed, which could not be changed due to the retrospective nature of the study, the lack of data for several interpatient differences potentially affecting flap tissue perfusion, such as the length and diameter of the flap pedicle, should be noted. Furthermore, it cannot be ruled out that higher and more variable norepinephrine doses might nevertheless be associated with flap tissue perfusion. Moreover, the applicability of the findings observed for this study cohort to a less sedated patient cohort remains uncertain.

This retrospective pilot study was conducted to analyze the effect of intravenously administered norepinephrine on early postoperative tissue perfusion of RFFFs in the head and neck region during continuous flap monitoring with the O2C analysis system using attached surface probes. The study revealed that tissue perfusion of RFFFs in terms of blood flow and hemoglobin oxygen saturation was generally not influenced by intravenously administered norepinephrine. In terms of clinical implications, the lack of an influence of intravenously administered norepinephrine on microvascular flap tissue perfusion supports the reliability of predefined absolute thresholds used to detect vascular flap compromise during continuous postoperative monitoring of RFFFs with the O2C analysis system and may mitigate concerns about the use of vasopressors in the postoperative hemodynamic management of patients undergoing microvascular head and neck reconstruction with RFFFs. Further additional studies with a substantial number of patients and an extended interval of flap monitoring upon controlling for confounding factors are required.

## Conclusion

This study showed that early postoperative tissue perfusion of RFFFs in terms of blood flow and hemoglobin oxygen saturation was in general not influenced by intravenously administered norepinephrine. This may support the reliability of absolute thresholds used to detect vascular flap compromise during continuous postoperative monitoring of RFFFs with the O2C analysis system. However, additional studies with substantial sample sizes and extended monitoring intervals are needed to confirm these findings. 

## Data Availability

The datasets generated and analyzed during the current study are under further analysis and are available from the corresponding author on reasonable request.

## Abbreviations

RFFF: Radial free forearm flaps
O2C: Oxygen-to-see
